# Prediction Distribution Model of Moisture Content in Laminated Wood Components

**DOI:** 10.3390/polym16111453

**Published:** 2024-05-21

**Authors:** Panpan Tian, Jianhong Han, Shangjie Guo, Jun Di, Xia Han

**Affiliations:** 1 School of Civil Engineering and Architecture, Xinjiang University, Urumqi 830047, China; tianpanpan@xju.edu.cn (P.T.); 18863523979@163.com (S.G.); hanxiaixa@xju.edu.cn (X.H.); 2 School of Civil Engineering, Xinjiang Institute of Engineering, Urumqi 830023, China; 3 Xinjiang Architectural Design and Research Institute Co., Ltd., Urumqi 830002, China; dijun67@163.com

**Keywords:** laminate wood components, humidity field, moisture content distribution, absorption, desorption

## Abstract

Shrinkage cracks are some of the most common defects in timber structures obtained from woods with an uneven distribution of moisture content and are subject to external dynamic environmental changes. To accurately predict the changes in the moisture content of wood components at any time and position, this study first applied the principles of food drying and established a moisture field model for laminated wood based on the analogy between heat and humidity transfer. A model for predicting the moisture content of wood that considers time and spatial distribution was then proposed. Second, by collecting relevant experimental data and establishing a finite element analysis model, three moisture absorption conditions (0–9.95%, 0–13.65%, and 0–17.91%) and four desorption conditions (34–5.5%, 28–8.3%, 31–11.8%, and 25.5–15.9%) were analyzed. In the moisture absorption comparison, the time needed to reach 95% equilibrium moisture content was 2.43 days, 4.07 days, and 6.32 days. The rate at which the internal components reached equilibrium moisture content exceeded 10 days. The temporal and spatial distribution of wood moisture content revealed the correctness of the proposed wood moisture field model. Finally, the moisture content prediction model was applied in the order of characteristic equation solutions, moisture content gradient difference, and laminated wood size. The results revealed that the established humidity field model can predict the wood moisture content and how it changes over time and in space. Notably, 1–2 orders for the solution of the characteristic equation are recommended when applying the prediction model. The greater the difference in moisture content, the faster the equilibrium moisture content is reached. The moisture content varies greatly based on the component size and position. Notably, the influence of moisture gradient and wood size on the average wood moisture content cannot be ignored.

## 1. Introduction

Wood is a low-carbon [1], hygroscopic material; hence, it is always subject to absorption and desorption with the continuous change in the surrounding environment before ultimately reaching a balance between the internal and external humidity. Moisture content is a crucial wood parameter [2], as it directly affects the mechanical properties [3], decay law [4,5], and shrinkage cracking of wood [6,7,8]. Wood is widely used in the furniture and construction industries due to its renewability, environmental friendliness, and comfort properties. However, the scarcity of analytical models for predicting the moisture field of wood is a recognized problem in the field. Therefore, an analytical model of the wood moisture field for accurately and quickly predicting the changes in wood moisture content over time, as well as its spatial distribution, is required.

Most scholars have focused on experimental testing to investigate the variation in wood moisture content over time. Baranski et al. [9] analyzed the influence of the impregnation of pine wood (*Pinus sylvestris* L.) samples on the changes in electrical resistance and the accuracy of moisture content measurements. Chomeharn et al. [10] proposed that the moisture content of wood varies sinusoidally with external humidity; however, the latter is secondary to the change in relative humidity. Yang et al. [11] tested the moisture content of poplar wood after a cycling test in an environment with sinusoidal changes in relative humidity. The findings corroborated those of Chomeharn et al. [10]. Time [12,13] placed Norwegian Chinese fir in an environment with a temperature of 25 °C and cycles of relative humidity ranging from 54 to 94% to investigate the change in water content. Thereafter, the author established a mathematical model of water transport based on Fick’s law and water vapor pressure. Fan et al. [14] demonstrated that the moisture content of wood varies exponentially with time under air drying. Jiang et al. [15] conducted numerical simulations of temperature and water diffusion fluctuations during the drying of birch wood. Some scholars have made valuable distribution theoretical advances in moisture content. Arends et al. [16] believed that the change in wood moisture content was related to the frequency of the environmental humidity cycle and established the diffusion equation of water. Baronas et al. [17] proposed a two-dimensional numerical model to measure moisture transport in wood and used computer simulation to predict the moisture content of wood and its change rule. The correctness of the model was verified through analysis and comparison. Duan et al. [18] proposed a highly effective method for predicting wood moisture content based on terahertz time-domain spectroscopy.

Similarly, regarding the spatial distribution of moisture content within the wood, most scholars have focused on experimental measurement and numerical simulation. Chen et al. [19] measured the moisture content distribution in the cross-sections of six Chinese fir specimens of different sizes through environmental experiments. Fu et al. [20] demonstrated a method for measuring moisture content and shrinkage strain during electrochemical wood drying and the feasibility of the electrochemical method. Fukui et al. [21] measured the moisture content of three types of green cork logs based on vibration, namely sugi (*Cryptomeria japonica*), todomatsu (*Abies sachalinensis*), and hinoki (*Chamaecyparis obtusa*). Awais et al. [22] used near-infrared hyperspectral imaging to predict the moisture distribution on the surface of wood at a macroscopic scale. Fragiacomo et al. [23] used an ABAQUS model to calculate the moisture content of a wood-member section based on the temperature and humidity data of different environments in Europe, with the cross-section size and protective layer as the variables. Lü et al. [24] used an X-ray method to calculate the spatial distribution of wood moisture content. Kang et al. [25] studied the radial water content distribution of a larch section during RF/vacuum drying via the circumferential slice method and concluded that cracking was mainly related to the radial distribution of water content. Some scholars have studied the spatial distribution of moisture content inside wood through fitting. Jia et al. [26] and Zhan et al. [27] studied wood drying, and the moisture content of wood exhibited a distribution law of high inside the cross-section and low outside, which resembled the distribution form of the quadratic polynomial. The results of the cross-sectional water content distribution fitted by Chen [28] were similar to those of Jia et al. [26] and Zhan et al. [27].

However, the research on predicting wood moisture content is deficient in three aspects. First, the distribution of moisture content at the level of wooden structural components has not been sufficiently analyzed in the literature, and no method to accurately predict the changes in the moisture content of wooden components at any time and position has been presented. Second, the moisture content in the above studies has mostly been measured in the water equilibrium state before and after cycling, and the analysis of wood response behavior throughout the entire cycling process is relatively limited. Third, the research has mainly been based on experiments and numerical simulations, and no theoretical prediction model has been proposed.

To address the shortcomings in the literature, this paper proposes an analytical humidity field model for laminated wood components to predict the moisture distribution in wood at any time and position. The remainder of this manuscript is structured as follows: Section 2 introduces the analogical relationship between heat transfer and humidity transfer, which is the theoretical basis for the proposed model. Section 3 establishes a universal model for the humidity field of wood components based on the principles of food drying and builds on Section 2. Section 4 validates the prediction model from two aspects based on relevant experimental and numerical simulation results: the variation in moisture content over time and its spatial distribution. Section 5 further applies the model by considering the selection of model parameters, the impact of moisture content, and the component dimensions.

## 2. Analogy of Unsteady Heat Transfer and Humidity Transfer

The humidity transfer module in the finite element analysis software for wood materials and components is currently incomplete, unlike the temperature transfer module. Furthermore, heat transfer and humidity transfer in wood components are similar phenomena. To facilitate the analysis of a temperature field and humidity field model, the parameter analogy of instantaneous heat transfer and humidity transfer should be determined. Once the analogy parameters and governing equation are determined, the problem of humidity transfer can be solved. The parameters and governing equations analogies of unsteady heat transfer and humidity transfer are listed in Table 1 and Table 2, respectively.

## 3. Establishment of Humidity Field Distribution Model

The theoretical basis for the humidity field model in this study is built on the principles of food drying. There are three main reasons for analyzing changes in wood moisture content during food drying. First, wood is a plant fiber, similar to food items such as apples and pineapples. Second, moisture content is the most important parameter for wood as well as food drying, especially changes in moisture content. Furthermore, changes in moisture during food drying as well as wood moisture content are influenced by external factors.

### 3.1. General Analytical Solution of Humidity Transfer Control Equation

Assume *D* and *S* are constants based on the analogy relationship between heat transfer and humidity transfer described in the previous section. Introduce the relative water content *ϕ* into the governing equations of plates, cylinders, and spheres, as shown in Equation (1).

(1) $$\begin{array}{l} \frac{1}{D}\frac{\partial W(\rho ,t)}{\partial t}=\left\{\begin{array}{l} \frac{\partial^{2}\phi }{\partial \rho^{2}}\\ \frac{\partial^{2}\phi }{\partial \rho^{2}}+\frac{1}{\rho }\frac{\partial \phi }{\partial r}\\ \frac{\partial^{2}\phi }{\partial \rho^{2}}+\frac{2}{\rho }\frac{\partial \phi }{\partial r} \end{array}\right. \end{array}$$

where *ρ* = *r*/*R* is the ratio of the radial distance *r* from the position of any point to the center and half of the radial length *R*.

The initial and boundary conditions are expressed in Equations (2) and (3).

(2) $$t=0,\;\;\;W\left(\rho ,0\right)=W_{0},\;\;\;\phi \left(\rho ,0\right)=1$$

(3) $$-\frac{\partial W}{\partial \rho }{|}_{\rho =1}=\frac{S}{D}(W_{e}-W_{s})$$

Predicting the temporal and spatial distribution of moisture in wood components using the humidity distribution model is critical. Based on the analogy relationship between the temperature field and the humidity fields in the previous section and the results of Adebiyi [29], the general solution is obtained, as shown in Equation (4).

(4) $$\phi (\rho ,t)=\sum_{n=1}^{\infty }\frac{2Bi}{\left[\mu_{n}^{2}+Bi^{2}+2\nu Bi\right]}\left[\frac{\rho^{\nu }J_{-\nu }(\rho \mu_{n})}{J_{-\nu }(\mu_{n})}\right]\exp(-\mu_{n}^{2}Fo)$$

where *J* is the first type of Bessel function, and *μ_n_* is the solution to the characteristic equation, as shown in Equation (5).

(5) $$\frac{J_{-\nu }(\mu_{n})}{J_{-(\nu -1)}(\mu_{n})}=\mu_{n}/Bi$$

where *ν* is the Bessel function series of the first type. Its values are 1/2, 0, and 2 for the plane slab, cylinder, and shell, respectively [29].

The solution of the Bessel function of the first type is expressed in Equations (6)–(10).

(6) $$J_{n}(x)=\sum_{m=0}^{\infty }\frac{{\left(-1\right)}^{m}}{m!\;\;\Gamma (n+m+1)}{\left(\frac{x}{2}\right)}^{n+2m}$$

(7) $$J_{0}(x)=\sum_{m=0}^{\infty }\frac{{\left(-1\right)}^{m}}{{\left(m!\right)}^{2}}{\left(\frac{x}{2}\right)}^{2m}$$

(8) $$J_{1}(x)=\sum_{m=0}^{\infty }\frac{{\left(-1\right)}^{m}}{m!(m+1)!}{\left(\frac{x}{2}\right)}^{2m+1}$$

(9) $$J_{-1/2}(x)=\sqrt{\frac{2}{\pi }}\frac{\cos(x)}{\sqrt{x}}$$

(10) $$J_{1/2}(x)=\sqrt{\frac{2}{\pi }}\frac{\sin(x)}{\sqrt{x}}$$

### 3.2. Distribution of Moisture Content in Time

Equation (11) shows the characteristic values obtained by substituting the series of Bessel functions of the first type for laminate components *ν* = 1/2 into Equation (4).

(11) $$\frac{J_{-1/2}(\mu_{n})}{J_{1/2}(\mu_{n})}=\cot(\mu_{n})=\mu_{n}/Bi;\;\;\mu_{n}\tan(\mu_{n})=Bi$$

The change in the moisture content of the laminate components over time can then be obtained, as shown in Equations (12) and (13).

(12) $$\phi (\rho ,t)=\sum_{n=1}^{\infty }\frac{2Bi}{\left[\mu_{n}^{2}+Bi^{2}+Bi\right]}\left[\frac{\sqrt{\rho }\sqrt{2/\pi }\frac{\cos(\rho \mu_{n})}{\sqrt{\rho \mu_{n}}}}{\sqrt{2/\pi }\frac{\cos(\mu_{n})}{\sqrt{\mu_{n}}}}\right]\exp(-\mu_{n}^{2}Fo)$$

(13) $$\phi (\rho ,t)=\sum_{n=1}^{\infty }\frac{2Bi\cos(\rho \mu_{n})}{\left[\mu_{n}^{2}+Bi^{2}+Bi]\cos(\mu_{n}\right)}\exp(-\mu_{n}^{2}Fo)$$

### 3.3. Spatial Distribution of Moisture Content

By substituting the boundary condition *ϕ* (0, *t*) of the moisture content of the neutral layer into Equation (12), the moisture content of the neutral layer can be obtained, as shown in Equation (14).

(14) $$\Phi (t)=\sum_{n=1}^{\infty }\frac{2Bi}{\left[\mu_{n}^{2}+Bi^{2}+Bi]\cos(\mu_{n}\right)}\exp(-\mu_{n}^{2}Fo)$$

Substituting the boundary condition *ϕ* (1, *t*) of surface moisture content into Equation (12), the surface moisture content can be obtained, as shown in Equation (15).

(15) $$\Phi_{s}(t)=\sum_{n=1}^{\infty }\frac{2Bi}{\left[\mu_{n}^{2}+Bi^{2}+Bi\right]}\exp(-\mu_{n}^{2}Fo)$$

The average moisture content is shown in Equations (16) and (17).

(16) $$\bar{\phi }(t)=\frac{1}{R}\int_{0}^{R}\phi (r,t)dr=\sum_{n=1}^{\infty }\frac{2Bi\exp(-\mu_{n}^{2}Fo)}{\left[\mu_{n}^{2}+Bi^{2}+Bi]\cos(\mu_{n}\right)}\int_{0}^{1}\cos(\rho \mu_{n})d\rho$$

(17) $$\bar{\phi }(t)=\sum_{n=1}^{\infty }\frac{2Bi}{\left[\mu_{n}^{2}+Bi^{2}+Bi]\cos(\mu_{n}\right)}\frac{\sin(\mu_{n})}{\mu_{n}}\exp(-\mu_{n}^{2}Fo)$$

Equations (18) and (19) show *μ_n_* and the average moisture content when *Bi* > 100.

(18) $$\mu_{n}=(2n-1)\frac{\pi }{2},n=1,2,\ldots \ldots \cot(\mu_{n})=0$$

(19) $$\bar{\varphi }(t)=\frac{8}{\pi^{2}}\sum_{n=1}^{\infty }\frac{1}{{(2n-1)}^{2}}\exp\left[-{\left(2n-1\right)}^{2}\frac{\pi^{2}}{4}Fo\right]$$

## 4. Validation of Humidity Field Distribution Model

The distribution of moisture in time and space, as shown in Equations (11)–(19), was verified to prove the correctness of applying the drying theory of plate food (bread slice, bacon slice, etc.) to the humidity field model of laminated wood components. This section compares and verifies the moisture field model of laminated wood components proposed in this paper using experimental data obtained from relevant scholars and finite element numerical simulation results.

### 4.1. Related Test

Simpson conducted moisture absorption and desorption tests on laminate wood [30,31]. The test data in this section were mainly from the test data of Simpson. Simpson’s moisture absorption and desorption tests were conducted on 76 mm × 146 mm × 12.7 mm and 50 mm × 50 mm × 25 mm boards, respectively.

In the desorption test, Simpson processed the wooden logs into 76 mm × 25 mm × 900 mm longboards, which were reworked into a series of end-matched specimens (102 mm and 13 mm along the wood grain). All specimens reached the equilibrium moisture content at different relative humidities (53%, 70%, 83%, and 92%). Aluminum paint was sealed around the 102 mm specimen to limit humidity transfer in the radial direction. Because the diffusion rate of the 13 mm specimen on the cross-section is much higher than the radial diffusion rate of the 102 mm specimen, the 13 mm specimen was used to estimate the equilibrium moisture content of the 102 mm specimen to shorten the time to equilibrium. The balanced specimens were divided into four groups and placed under the same temperature (43 °C) but different humidity conditions (53%, 70%, 83%, and 92%) for desorption. The 76 mm × 102 mm specimen was also included to confirm the moisture content gradient. These specimens were taken out regularly, and the 50 mm × 50 mm × 25 mm specimens cut from the middle were cut into 10 slices with a thickness difference of approximately 1.25 mm. The slices were sealed in a polyethylene bag, and all the specimens were weighed uniformly after slicing. After desorption, all specimens were placed in a 30% relative humidity environment. After the equilibrium moisture content was reached, relative humidities of 53%, 70%, 83%, and 92% were reached at a temperature of 43 °C through absorption. In the second absorption test, gradient specimens were not included. The main experimental parameters are listed in Table 3.

In the absorption test, the initial moisture content *W*_0_ of all eight groups was 0%, and the equilibrium moisture content *W*_e_ was 2.69%, 5.06%, 7.50%, 9.95%, 12.16%, 13.65%, 16.22%, and 17.91%. The water diffusion coefficient *D* was calculated according to the regression formula [30,31], which was 0.377 mm^2^/h, 0.505 mm^2^/h, 0.685 mm^2^/h, 0.918 mm^2^/h, 1.21 mm^2^/h, 1.47 mm^2^/h, 2.01 mm^2^/h, and 2.48 mm^2^/h, respectively. The surface humidity divergence coefficient *S* was calculated based on half of the total time to reach equilibrium moisture content, which was 1.88 m/h, 2.01 m/h, 2.57 m/h, 3.39 m/h, 3.65 m/h, 4.12 m/h, 5.35 m/h, and 6.61 m/h, respectively.

In the desorption test, the initial moisture content *W*_0_ of all four groups was 34%, 28%, 31%, and 25.5%, and the equilibrium moisture content *W*_e_ was 5.5%, 8.3%, 11.8%, and 15.9%, respectively. The water diffusion coefficient *D* was 1.3805 mm^2^/h, 1.3553 mm^2^/h, 1.4069 mm^2^/h, and 1.3956 mm^2^/h, respectively, and the surface humidity divergence coefficient *S* was 2.3456 m/h, 3.3653 m/h, 3.2301 m/h, and 0.6234 m/h, respectively.

### 4.2. Finite Element Analysis

**Figure 1:**
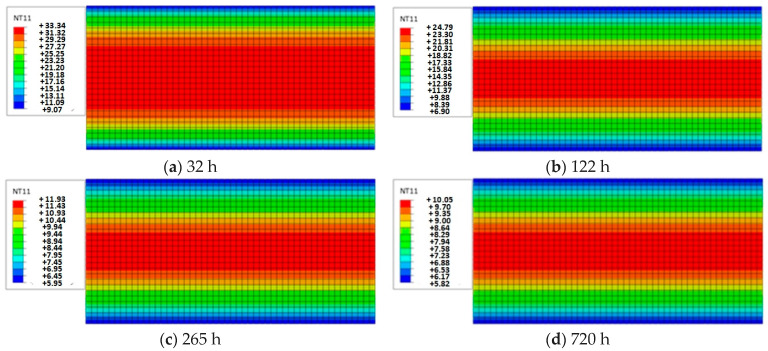
Establishment of finite element model and numerical simulation results.

Sun’s results were used in the finite element modeling method [32]. The finite element simulation mainly utilizes the temperature field transfer module of the large-scale finite element analysis software ABAQUS 6.14. All parameters of the model were established based on the parameters provided in the work of Simpson regarding moisture absorption and desorption (Table 3). To achieve both computational speed and accuracy, the length direction of the laminate was taken as 1/4 of the length of the test component in the finite element model, and the mesh size was approximately 1 mm. The three-dimensional, eight-node heat transfer unit DC3D8 was selected for humidity transfer analysis. Due to the sealing aluminum paint used around the wood components, the surface coefficient of divergence *S* was set to 0. Only the upper and lower surfaces transfer humidity. Figure 1a,b show the finite element model. The main parameters and material parameters of the model are listed in Table 4 [19].

The above FEM simulation method was used to conduct a numerical simulation analysis of the 12 sets of tests listed in Table 3. Due to space limitations, the 9th group of trials (34–5.5%) in Table 3 was taken as an example owing to the moisture content distribution in each cross-section along the length direction being consistent. Figure 1a–d show that the moisture distribution in any section changes with time (32 h, 122 h, 265 h, and 720 h).

The modeling results in Figure 1 indicate that the moisture content outside the specimen changes rapidly with time, and the equilibrium moisture content can be reached in a few hours. However, the internal moisture content changes very slowly; even at the 720th hour, the equilibrium moisture content is not fully reached.

### 4.3. Moisture Content Changes over Time

**Figure 2:**
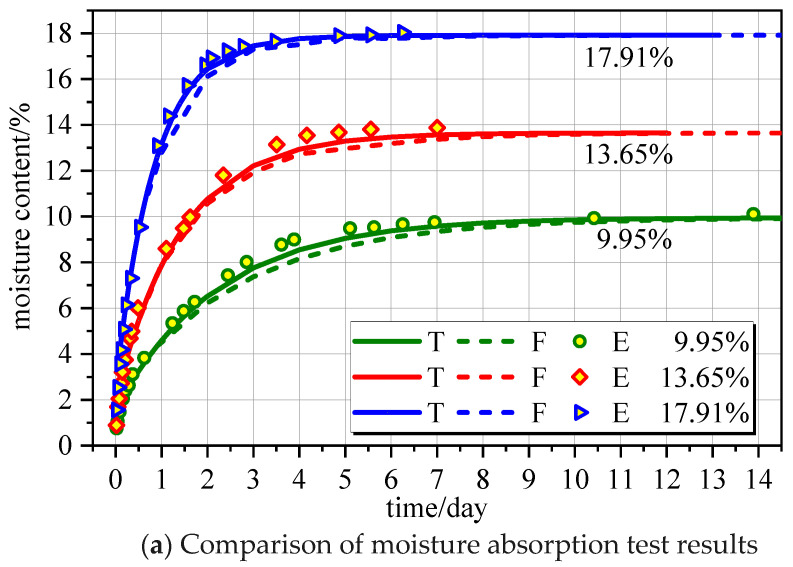

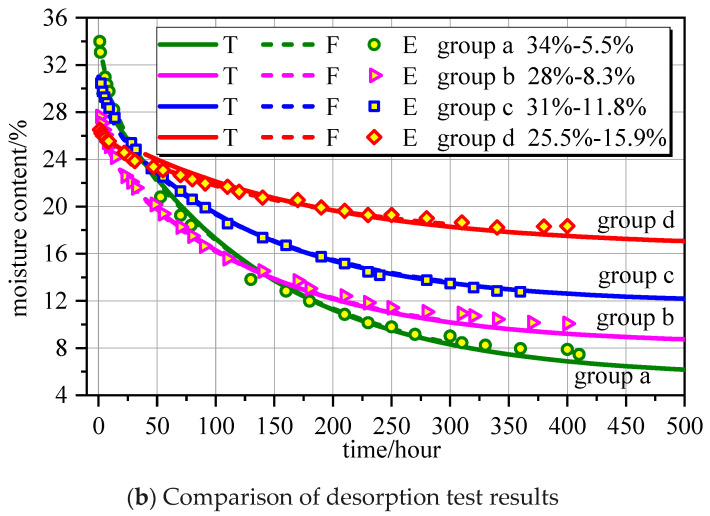
Verification of the calculation model of water content change over time.

The extracted moisture absorption test data [30] and desorption test data [31], as well as the finite element simulation results and theoretical results calculated from Equation (13), are shown in Figure 2. Notably, T, F, and E represent the theoretical calculation formula, finite element simulation results, and experiment data, respectively.

Figure 2 shows that the theoretical model of the moisture content of wood components with time is in good agreement with the test data and finite element results. The greater the moisture content difference (*W*_e_ − *W*_0_), the faster the equilibrium moisture content is reached. In the moisture absorption comparison, specimens with equilibrium moisture content ranged from high to low, and the time needed to reach 95% equilibrium moisture content was 2.43 days, 4.07 days, and 6.32 days. In the desorption comparison, the greater the moisture content difference (*W*_e_ − *W*_0_), the faster the equilibrium moisture content was reached, which is consistent with the moisture absorption findings. Notably, the characteristic equation root *μ_n_* of the first type of the Bessel function *J* takes the first term.

### 4.4. Moisture Content Changes in Space

**Figure 3:**
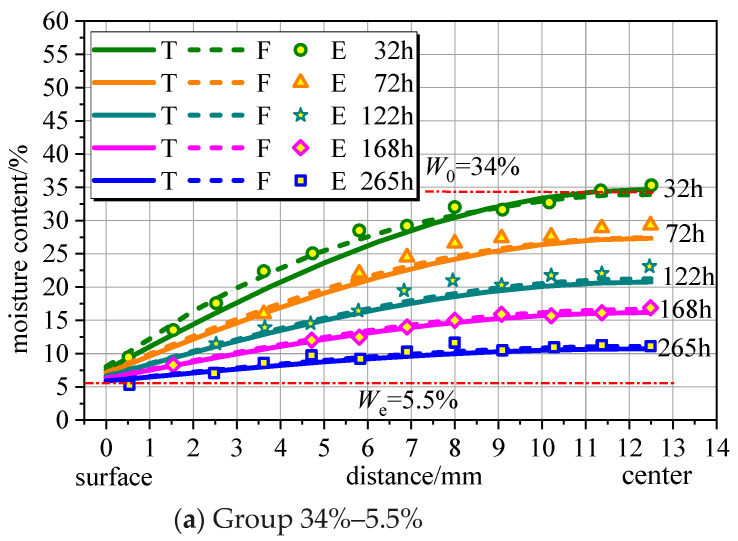

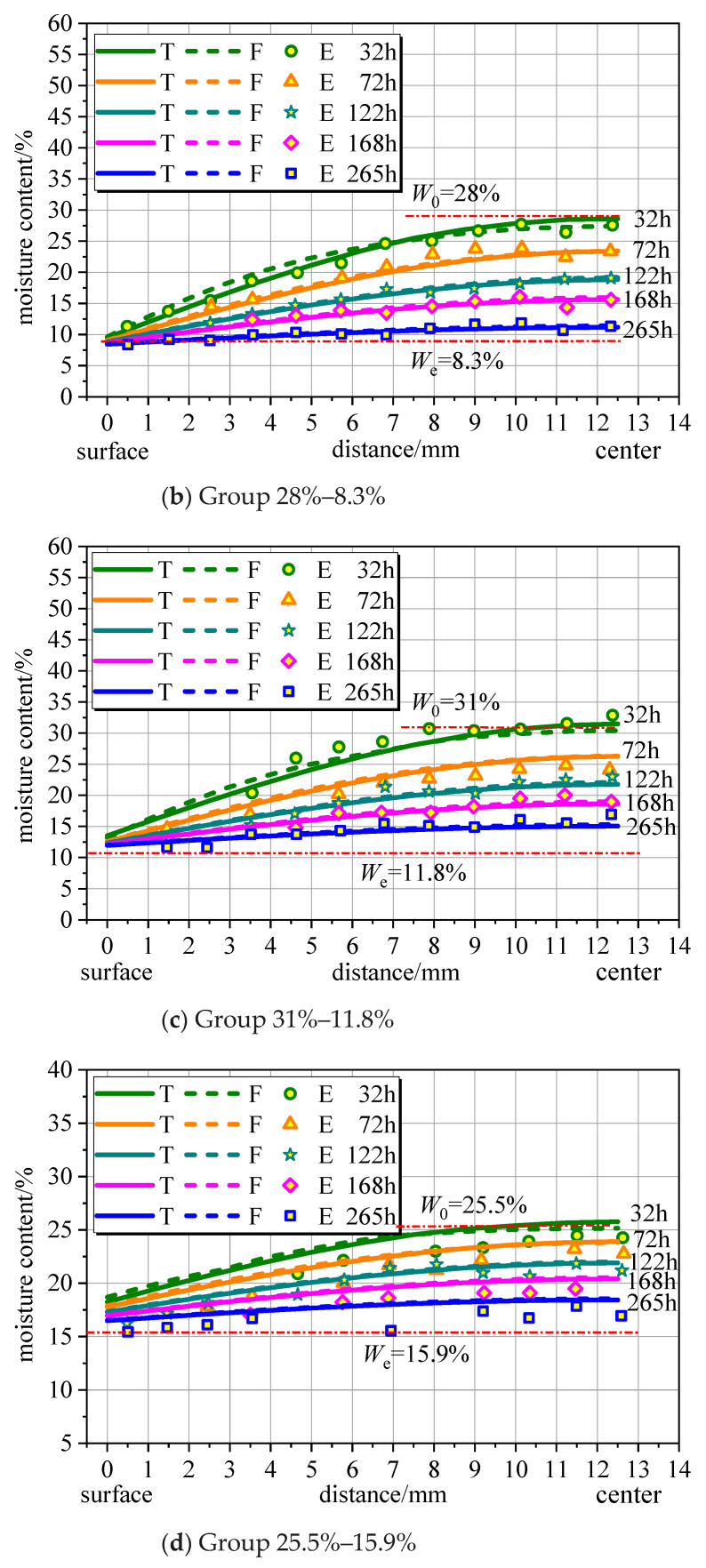
Verification of the calculation model of moisture content change in space.

To verify the change in moisture content in space, four groups of desorption data (34–5.5%, 28–8.3%, 31–11.8%, and 25.5–15.9%) were selected for analysis. Thereafter, several typical time points were selected from each group of data and summarized. The desorption test data [31], finite element simulation results, and theoretical results calculated from Equations (16)–(19) are drawn in Figure 3. Notably, T, F, and E represent the theoretical calculation formula, finite element simulation results, and experiment data, respectively.

Figure 3 shows that the theoretical model of the moisture content of wood components in space is in good agreement with the test data and finite element results. Initially, the rate at which the equilibrium moisture content is reached on the outside is much greater than that on the inside of the component. Even after more than 10 days, the equilibrium moisture content inside the wood had not been reached. This conclusion regarding the distribution of moisture content over time is consistent. Notably, the first term of the characteristic equation root *μ_n_* of the Bessel function *J* was taken in this research.

## 5. Discussion

### 5.1. Solution of the Characteristic Equation

**Figure 4:**
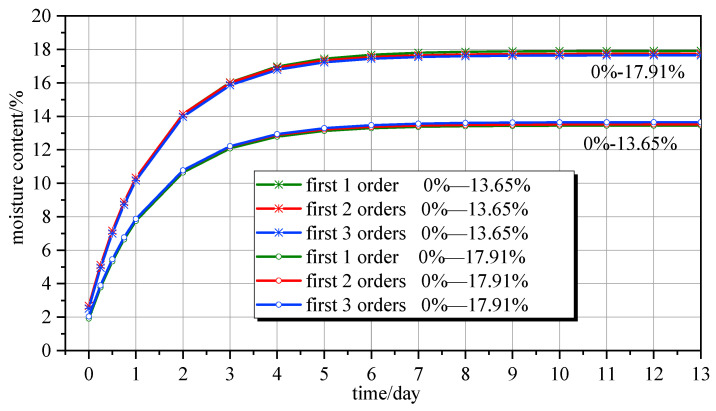
Influence of the order of *μ_n_* on the accuracy of the average water content.

The calculation formula for predicting water content, whether it is distributed in time or space, involves an important calculation parameter, *μ_n_*, which affects the accuracy of the model calculation results. This research considered two sets of data, namely 0–17.91% and 0–13.65%, as examples. The parameters *R*, *D*, and *S*, among others, were taken from references [30] and [31] and denote the first 1, 2, and 3 orders of *μ_n_*, respectively. The average moisture content over time was calculated, as shown in Figure 4. The first three orders of *μ_n_* for group 0–17.91% were 1.4834, 4.4550, and 7.440, respectively. Likewise, the first three orders of *μ_n_* for group 0–13.65% were 1.4874, 4.4665, and 7.4572, respectively.

Figure 4 shows that regardless of the first 1, 2, and 3 orders of *μ_n_* being taken in the average moisture content prediction calculation formula, the curves largely overlapped. This is attributable to the order of *μ_n_* affecting the accuracy of the prediction calculation model. For laminated wooden components, the first 1 order has good calculation accuracy, so it coincides with the curve of the first 3 orders. Specifically, taking the first 1 order increases accuracy within 3%. For convenience of calculation, the first 1–2 orders for laminated wood components were taken in further calculations.

### 5.2. Moisture Gradient

**Figure 5:**
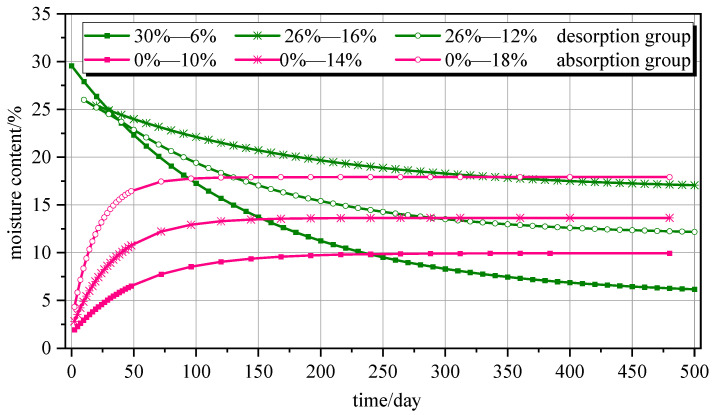
Influence of moisture content differences on average moisture content.

The moisture content difference is the driving force behind moisture transfer in wood components. Three different moisture content differences were selected for moisture absorption and desorption and substituted into Equation (17), and the calculation results are shown in Figure 5.

Figure 5 shows that regardless of moisture absorption or desorption, the larger the moisture content difference, the faster the equilibrium moisture content is reached. In the moisture absorption group, the time required to reach the equilibrium moisture content in the specimens was 95 h, 148 h, and 242 h. The main reason for the change in wood moisture content is the driving force provided by the moisture gradient. The larger the moisture content gradient, the greater the driving force. Therefore, the target moisture content of the external environment where the wood is located can be reached earlier. Notably, the results from the desorption group and the hygroscopic group were consistent.

### 5.3. Component Dimensions

**Figure 6:**
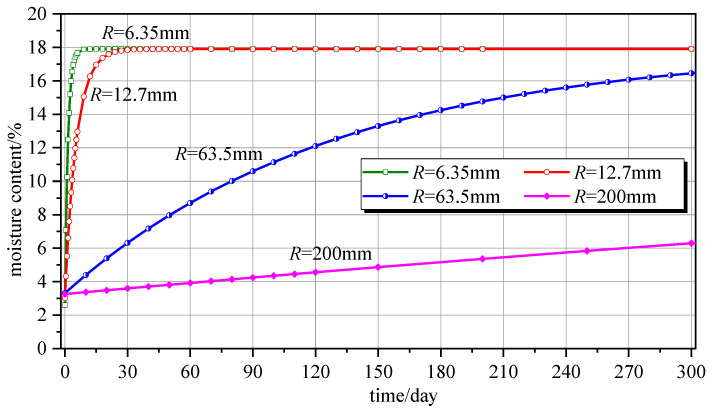
Influence of component dimensions on average moisture content.

The most critical parameter in wood components is dimension. Equation (17) was used to calculate the time required for laminated components with thicknesses of 6.35 mm, 12.7 mm, 63.5 mm, and 200 mm to reach the equilibrium moisture content (18%) from 0%, as shown in Figure 6.

The time requirements for laminated components with thicknesses of 6.35 mm, 12.7 mm, 63.5 mm, and 200 mm were 3.9 days, 35 days, over 800 days, and thousands of days, respectively. Noticeably, the moisture absorption and desorption rates of the component are related to the dimensions of the component. A component with *R* = 200 mm requires thousands of days to reach the equilibrium moisture content, and this duration is higher for larger wood components. The thicker the laminated components, the longer the duration before the equilibrium moisture content is reached and the slower the moisture absorption and desorption. For large wood components, a long duration is required to fully achieve equilibrium moisture content for two main reasons. On the one hand, the path of water transfer from the surface to the interior of the wood component is significantly longer. On the other hand, the transmission rate of moisture content is nonlinear, and the rate at which the equilibrium moisture content is reached is slow toward the interior of the wood component.

## 6. Conclusions

By applying the analogy between heat transfer and humidity transfer, this research introduced the theory of food drying, established a humidity field model for laminated wood components, and optimized the formula for predicting moisture content. Furthermore, the model and prediction formula were verified through relevant experimental data and finite element analysis. The following are the main conclusions:

(1) The theory of food drying can be applied to the calculation of wood moisture content.
(2) The established moisture content model and optimized calculation formula can be used to determine the moisture content of laminated wood components at any time and position, which can improve the convenience of future experiments and wood inspection.
(3) Meanwhile, the speed at which the outer side of the wooden component reaches equilibrium moisture content is much greater than that of the inner side. Even after hundreds of hours, the internal moisture content of the wood does not reach equilibrium.
(4) Regarding the humidity field model or moisture content prediction formula for laminated wood components, the first one or two orders are recommended for the root of the characteristic equation to meet the accuracy requirements.
(5) Regardless of moisture absorption or desorption, the greater the difference in moisture content, the greater the speed at which equilibrium moisture content is achieved.
(6) The distribution of moisture content varies greatly among different component sizes and at different positions of the same size.

This study can provide a theoretical basis and calculation formulas for the humidity field prediction of laminated wood components. However, this research is limited to laminated wood components, and the calculation of cylindrical wood components will be studied in the future.

## Figures and Tables

**Table 1:** Parameters analogy of unsteady heat and humidity transfer.

Heat Transfer		Humidity Transfer	
Temperature	*T*(K)	Moisture content	*W*(%)
Initial temperature	*T_i_*	Initial moisture content	*W_i_*
Ambient temperature	*T*_e_	Equilibrium moisture content	*W*_e_
Surface temperature of the object	*T*_s_	Surface moisture content	*W*_s_
Dimensionless relative temperature	$\theta =\frac{T-T_{e}}{T_{i}-T_{e}}$	Dimensionless relative moisture content	$\phi =\frac{W-W_{e}}{W_{i}-W_{e}}$
Thermal conductivity	*α*(m^2^/s)	Water diffusion coefficient	*D*(m^2^/s)
Surface heat exchange coefficient	*h*(W/m^2^/K)	Surface humidity divergence coefficient	*S*(m/s)
Biot number of heat transfer	$Bi_{T}=hL/\alpha$	Biot number of humidity transfer	$Bi_{W}=SL/D$
Fourier number	$Fo_{T}=\alpha t/L^{2}$	Fourier number	$Fo_{W}=Dt/L^{2}$

**Table 2:** Governing equations analogy of two-dimensional Fick’s law.

	Heat Transfer	Humidity Transfer
Cartesian coordinates	$\frac{\partial T}{\partial t}=\alpha \left(\frac{\partial^{2}T}{\partial x^{2}}+\frac{\partial^{2}T}{\partial y^{2}}+\frac{\partial^{2}T}{\partial z^{2}}\right)$	$\frac{\partial W}{\partial t}=D\left(\frac{\partial^{2}W}{\partial x^{2}}+\frac{\partial^{2}W}{\partial y^{2}}+\frac{\partial^{2}W}{\partial z^{2}}\right)$
Cylindrical coordinates	$\frac{1}{\alpha }\frac{\partial T}{\partial t}=\frac{1}{r}\frac{\partial }{\partial r}\left(r\frac{\partial T}{\partial r}\right)+\frac{\partial^{2}T}{\partial z^{2}}$	$\frac{1}{D}\frac{\partial W}{\partial t}=\frac{1}{r}\frac{\partial }{\partial r}\left(r\frac{\partial W}{\partial r}\right)+\frac{\partial^{2}W}{\partial z^{2}}$
Spherical coordinates	$\frac{1}{\alpha }\frac{\partial T}{\partial \theta }=\frac{1}{r^{2}}\frac{\partial }{\partial r}\left(r^{2}\frac{\partial T}{\partial r}\right)+\frac{1}{r^{2}\sin^{2}\phi }\left(\frac{\partial^{2}T}{\partial \phi^{2}}\right)$	$\frac{1}{D}\frac{\partial W}{\partial \theta }=\frac{1}{r^{2}}\frac{\partial }{\partial r}\left(r^{2}\frac{\partial W}{\partial r}\right)+\frac{1}{r^{2}\sin^{2}\phi }\left(\frac{\partial^{2}W}{\partial \phi^{2}}\right)$

Note: *t* represents the time.

**Table 3:** Main parameters of the Simpson test.

Group	Specimen Thickness (mm)	*W*_0_ (%)	*W*_e_ (%)	Test Type	Specimen Type (mm × mm × mm)	*D* (mm^2^/h)	*S* (mm/h)	*Bi* (%)
1	2*R* = 12.7	0	0.0269	absorption	76 × 146 × 12.7	0.377	1.88	28.7663
2	2*R* = 12.7	0	0.0506	absorption	76 × 146 × 12.7	0.505	2.01	20.5863
3	2*R* = 12.7	0	0.0750	absorption	76 × 146 × 12.7	0.685	2.57	17.4727
4	2*R* = 12.7	0	0.0995	absorption	76 × 146 × 12.7	0.918	3.39	15.2670
5	2*R* = 12.7	0	0.1216	absorption	76 × 146 × 12.7	1.21	3.65	11.3061
6	2*R* = 12.7	0	0.1365	absorption	76 × 146 × 12.7	1.47	4.12	9.9475
7	2*R* = 12.7	0	0.1622	absorption	76 × 146 × 12.7	2.01	5.35	8.3676
8	2*R* = 12.7	0	0.1791	absorption	76 × 146 × 12.7	2.48	6.61	7.7873
a	2*R* = 25.0	0.34	0.055	desorption	50 × 50 × 25	1.3805	2.3456	21.2386
b	2*R* = 25.0	0.28	0.083	desorption	50 × 50 × 25	1.3553	3.3653	31.0376
c	2*R* = 25.0	0.31	0.118	desorption	50 × 50 × 25	1.4069	3.2301	28.6983
d	2*R* = 25.0	0.255	0.159	desorption	50 × 50 × 25	1.3956	0.6234	5.5831

Note: 1. Moisture absorption test group specimen half thickness *R* = 6.35 mm. 2. Moisture desorption test group specimen half thickness *R* = 12.5 mm. 3. This study does not provide the test values of *D* and *S*. *D* is calculated according to the regression provided by the paper, and *S* is inversely calculated according to *t*_0.5_.

**Table 4:** Parameters of material.

Directions	Elastic Modulus (MPa)	Compressive Strength (MPa)	Tensile Strength (MPa)	Shrinkage/Swelling Coefficient (%)
R	*E*_R_ = 1048 (16.1%)	*f*_c,R_ = 3.07 (12.23%)	*f*_t,R_ = 3.07 (12.23%)	*α*_R_ = 0.139
T	*E*_T_ = 594 (22.7%)	*f*_c,T_ = 2.67 (13.44%)	*f*_t,T_ = 2.67 (13.44%)	*α*_T_ = 0.255
L	*E*_L_ = 12,888 (6.9%)	*f*_c,L_ = 36.25 (10.43%)	*f*_t,L_ = 36.25 (10.43%)	*α*_L_ = 0.019
Directions	Poisson ratio	Shear modulus (MPa)		
RT	*v*_RT_ = 0.43	*G*_RT_ = 232		
RL	*v*_RL_ = 0.03	*G*_RT_ = 967		
TL	*v*_TL_ = 0.02	*G*_RT_ = 773		

## Data Availability

The data are unavailable due to privacy or ethical restrictions.

## Nomenclature

*r*(mm)	The radial distance from the position of any point to the center
*R*(mm)	Half of the radial length *R*
*J*	The first kind of Bessel function
*μ_n_*	The solution of the characteristic equation
*ν*	The Bessel function series of the first type
*T*(K)	Temperature
*T_i_*(K)	Initial temperature
*T*_e_(K)	Ambient temperature
*T*_s_(K)	Surface temperature of the object
θ	Dimensionless relative temperature
*α*(m^2^/s)	Thermal conductivity
*h*(W/m^2^/K)	Surface heat exchange coefficient
$Bi_{T}$	Biot number of heat transfer
$Fo_{T}$	Fourier number
*W*(%)	Moisture content
*W_i_*(%)	Initial moisture content
*W*_e_(%)	Equilibrium moisture content
*W*_s_(%)	Surface moisture content
ϕ	Dimensionless relative moisture content
*D*(m^2^/s)	Water diffusion coefficient
*S*(m/s)	Surface humidity divergence coefficient
$Bi_{W}$	Biot number of humidity transfer
$Fo_{W}$	Fourier number
*t*	the time
*E*_R_, *E*_T_, *E*_L_	Radial/tangential/longitudinal elastic modulus
*f*_t,R_, *f*_t,T_, *f*_t,L_	Radial/tangential/longitudinal tensile strength in cross-section
*f*_c,R_, *f*_c,T_, *f*_c,L_	Radial/tangential/longitudinal compressive strength in cross-section
*α*_R_, *α*_T_, *α*_L_	Radial/tangential/longitudinal shrinkage and swelling coefficient
*v*_RT_, *v*_RL_, *v*_TL_	Poisson ratio of different directions
*G*_RT_, *G*_RL_, *G*_TL_	Shear modulus of different directions
